# A review of statistical methods for dietary pattern analysis

**DOI:** 10.1186/s12937-021-00692-7

**Published:** 2021-04-19

**Authors:** Junkang Zhao, Zhiyao Li, Qian Gao, Haifeng Zhao, Shuting Chen, Lun Huang, Wenjie Wang, Tong Wang

**Affiliations:** 1 Department of Health Statistics, School of Public Health, Shanxi Medical University, No.56 Xinjian South Road, Taiyuan, 030001 Shanxi province China; 2 Department of Nutrition & Food Hygiene, School of Public Health, Shanxi Medical University, No.56 Xinjian South Road, Taiyuan, 030001 Shanxi province China

**Keywords:** Dietary patterns, Dietary quality scores, Principal component analysis, Factor analysis, Clustering analysis, Treelet transform, Reduced rank regression, Data mining, Least absolute shrinkage and selection operator, Compositional data analysis

## Abstract

**Background:**

Dietary pattern analysis is a promising approach to understanding the complex relationship between diet and health. While many statistical methods exist, the literature predominantly focuses on classical methods such as dietary quality scores, principal component analysis, factor analysis, clustering analysis, and reduced rank regression. There are some emerging methods that have rarely or never been reviewed or discussed adequately.

**Methods:**

This paper presents a landscape review of the existing statistical methods used to derive dietary patterns, especially the finite mixture model, treelet transform, data mining, least absolute shrinkage and selection operator and compositional data analysis, in terms of their underlying concepts, advantages and disadvantages, and available software and packages for implementation.

**Results:**

While all statistical methods for dietary pattern analysis have unique features and serve distinct purposes, emerging methods warrant more attention. However, future research is needed to evaluate these emerging methods’ performance in terms of reproducibility, validity, and ability to predict different outcomes.

**Conclusion:**

Selection of the most appropriate method mainly depends on the research questions. As an evolving subject, there is always scope for deriving dietary patterns through new analytic methodologies.

## Background

Dietary intake, one of the essential factors that influence health, varies widely among individuals. The changes from the first Dietary Guidelines for Americans in 1980 to those in 2015 show that the focus of nutritional epidemiology has gradually shifted from single nutrients to dietary patterns, focusing on features of the entire diet [1]. There are several reasons for this shift [2]. First, each type of food contains multiple nutrients with complex interactions and latent cumulative relationships [3, 4]. Hence, it is not feasible to isolate and examine their separate effects on diseases [2]. Additionally, it is difficult to analyze the role of individual foods because a typical diet is characterized by a mixture of different foods with substitution effects, where an increase in the consumption of some foods will lead to a decrease in the consumption of others [5]. If we include all collected food items in an analytical model simultaneously, multicollinearity, due to the complex interactions and relationships among them, will make inferences about individual foods difficult [6]. Due to the growing recognition of the complexity of dietary intake and its interactions with health outcomes, research on the health effects of dietary patterns is necessary alongside that of individual nutrients [7]. Dietary patterns consider the complex interrelationships between different foods or nutrients as a whole, reflect individuals’ actual dietary habits, and provide more information to indicate when many nutrients are associated with diseases [1, 4]. Additionally, dietary patterns are more consistent over time and have a greater effect on health outcomes than individual nutrients [6]. Hence, dietary pattern analysis is considered a technology complementary to the study of single nutrients or food.

In the past few decades, statistical methods have emerged that make full use of dietary information collected across populations to create dietary patterns [2, 4, 8]. In nutritional epidemiology studies, regardless of the statistical method used for dietary pattern analysis, the goal is to explore the relationship between dietary patterns and health outcomes [2, 3]. From this perspective, evaluating a method depends not only on whether the dietary patterns derived by the method comprehensively reflect the dietary preferences but also on whether these patterns can predict diseases more accurately and promote health.

The majority of published reviews divide the statistical methods for dietary pattern analysis into three categories: investigator-driven, data-driven, and hybrid methods widely used in nutritional epidemiology [2, 3, 8–10]. Additionally, several emerging methods have been applied to dietary pattern analyses that are less often or never reviewed adequately. To demonstrate these methods more clearly, we classify the emerging methods based on the existing categories and add a new category.

Since the finite mixture model (FMM) is a model-based clustering method and treelet transform (TT) combines principal component analysis (PCA) and clustering algorithms in a one-step process, they are classified as data-driven methods. Data mining (DM) and least absolute shrinkage and selection operator (LASSO) consider health outcome in identifying dietary patterns and are therefore classified as hybrid methods. Compositional data analysis (CODA)—the latest addition in dietary pattern research—identifies dietary patterns by transforming dietary intake into log-ratios and is thus categorized separately due to the particularity of suitable data.

This paper provides an updated landscape review of these methods based on the underlying concepts, strengths, limitations, and software packages commonly used while paying particular attention to emerging methods. The subsequent content is introduced from the following aspects: (1) investigator-driven methods, containing various dietary scores and dietary indexes; (2) data-driven methods, comprising PCA, factor analysis, traditional cluster analysis (TCA), FMM, and TT; (3) hybrid methods, consisting of reduced rank regression (RRR), DM, and LASSO; (4) compositional data analysis, including compositional principal component coordinates, balance coordinates and principal balances. To conclude, we compare and evaluate these methods, identify the remaining methodological issues, and provide suggestions for future research.

## Investigator-driven methods

Investigator-driven methods are also called a priori approaches, and they include dietary scores and dietary indexes (collectively called dietary quality scores). These methods define dietary guidelines aligned with current nutritional knowledge or dietary recommendations that affect health as dietary patterns [9]. The foods or nutrients consumed by a person are scored based on some quality score (e.g., the Healthy Eating Index (HEI) shown in Table 1), and the results are summarized to produce dietary quality scores [12, 13]. Dietary quality scores measure the extent to which individuals adhere to dietary guidelines and recommendations to assess the population’s overall dietary quality and predict diseases [9, 13]. The classification of these scores is shown in Table 2.

**Table 1:** Components, point values, and standards for scoring of the Healthy Eating Index (HEI) [11]

Component	Maximum points	Standard for maximum score	Standard for a minimum score of zero
**Adequacy**			
Total Fruits	5	≥0.8 c equivalents/1000 kcal	No fruit
Whole Fruits	5	≥0.4 c equivalents/1000 kcal	No whole fruit
Total Vegetables	5	≥1.1 c equivalents/1000 kcal	No vegetables
Greens and Beans	5	≥0.2 c equivalents/1000 kcal	No dark green vegetables or beans and peas
Whole Grains	10	≥1.5 oz. equivalents/1000 kcal	No whole grains
Dairy	10	≥1.3 c equivalents/1000 kcal	No dairy
Total Protein Foods	5	≥2.5 oz. equivalents/1000 kcal	No protein foods
Seafood and Plant Proteins	5	≥0.8 c equivalents/1000 kcal	No seafood or plant proteins
Fatty Acids	10	(PUFAs^a^ + MUFAs^b^)/SFAs ^c^ ≥ 2.5	(PUFAs + MUFAs) / SFAs ≤1.2
**Moderation**			
Refined Grains	10	≤1.8 oz. equivalents/1000 kcal	≥4.3 oz. equivalents/1000 kcal
Sodium	10	≤1.1 g/1000 kcal	≥2.0 g/1000 kcal
Added Sugars	10	≤6.5% of energy	≥26% of energy
Saturated Fats	10	≤8% of energy	≥16% of energy

^a^*PUFAs* polyunsaturated fatty acids

^b^*MUFAs* monounsaturated fatty acids

^c^*SFAs* saturated fatty acids

**Table 2:** The dietary quality scores based on different classification methods

Classification Methods	Dietary Quality Scores
**Based on dietary standards** [8]	
Dietary guidelines	Healthy Eating Index (HEI) [11], Dietary Quality Index (DQI) [14], Alternative Healthy Eating Index (AHEI) [15], Dietary Lifestyle Index (DLI) [16]
Dietary recommendations	Recommended Food Score [17] and Composite Diet Score [18, 19]
**Based on dietary composition** [20]	
Nutrients	Dietary Quality (DQ) [21] and the Dietary Inflammatory Index (DII) [22]
Food or food group	Mediterranean Diet Score (MDS) [23], Mediterranean Diet Serving Score (MDSS) [24], and Healthy Food Index (HFI) [25]
Foods and nutrients	Diet Quality Index (DQI) [26], Healthy Eating Index (HEI) [11] and Dietary Approaches to Stop Hypertension (DASH) [27]
**Based on populations** [12]	Chinese Healthy Eating Index (CHEI) [28], Modified Food-Based Diet Quality Score for Japanese [29], Minimum Dietary Diversity for Women (MDD-W) [30], Mediterranean Diet Index for pregnant women (MDS-P) [31], Healthy Dietary Habits Score for Adolescents (HDHS-A) [32], Infant and Young Child Feeding Index (IYCFI) [33], and the Bone Mineral Density (BMD) diet score [34]

Recent studies on the relationship between dietary quality scores and health indicate that scores such as the HEI, Alternative Healthy Eating Index (AHEI) [15], Alternative Mediterranean Diet [35], and Dietary Approaches to Stop Hypertension (DASH) diet scores [27] are negatively correlated with the risk of death from cardiovascular disease, cancer, and all-cause mortality [36–40]. The last three dietary patterns were also recommended as easy and practical dietary plans for the public in the 2015 Dietary Guidelines for Americans [41]. Additionally, plant-based diets are receiving increasing attention because of their benefits to human health and environmental sustainability. Three plant-based diet indexes have been established in recent years: the total Plant-based Diet Index (PDI), Healthy Plant-based Diet Index (hPDI), and Unhealthy Plant-based Diet Index (uPDI) [42, 43]. Unlike other dietary quality scores, these plant-based dietary indexes focus on the quality of plant foods in the diet; all animal foods, including those animal foods known to promote health, are negatively scored when calculating the plant-based dietary indexes [44, 45]. Research has found that the higher the hPDI score, the lower the risk of coronary heart disease, type 2 diabetes, and all-cause mortality, whereas the uPDI shows the opposite trend [44–47].

Each component is individually scored and summed into a total score in the different scoring systems, but the total scores of different dietary quality scores vary greatly. Additionally, the total score can also be dichotomized but is less used [48, 49]. No research has been done to establish the preferable scoring system for specific situations [12]. It is important to consider the research purpose when applying dietary quality scores and that there is not necessarily a single diet plan to follow to achieve a healthy dietary pattern [9, 41].

### Advantages

The dietary guidelines and recommendations used to construct dietary quality scores are primarily based on scientific evidence from health and disease prevention studies. These scores can be used to describe overall dietary characteristics and repeat or compare results across populations. Many dietary quality scores have significant associations with disease and mortality outcomes. The total score is easy to understand and use, and the summing process is simpler than in other statistical methods for dietary pattern analysis.

### Disadvantages

The construction of the scores, the definition of dietary diversity, and interpretation of the guidelines are generally determined subjectively by the researchers [2]. Additionally, dietary scores cannot describe overall dietary patterns because they focus only on selected aspects of diet and do not consider the correlation of different dietary components [2, 13]. Finally, since a diet is usually multidimensional, the comprehensive dietary scores do not provide specific information on multiple foods, often leading to an unclear interpretation of intermediate scores. Individuals with a middle-range score likely have different nutritional compositions and dietary patterns [2, 9].

### Commonly available software and packages

No special program or package is required. Mainstream statistical analysis software, such as SAS, R, and STATA, are available.

## Data-driven methods

In nutritional epidemiological studies, data-driven methods refer to the dietary intake patterns derived from population data through data dimensionality reduction techniques. These methods use the existing data collected from food frequency questionnaires, 24-h recall questionnaires, or dietary records to obtain dietary patterns instead of defined dietary guidelines [2, 3, 50].

### Principal component analysis (PCA) and exploratory factor analysis (EFA)

PCA and EFA are the most commonly used methods in research on dietary patterns and, since they are based on similar mathematical concepts, they are discussed together in this section [3]. The PCA replaces a set of possibly correlating food groups with a new set of comprehensive indexes (principal components) that are uncorrelated and retain as much of the foods’ variance as possible. When deriving dietary patterns, it is common practice to pre-group food items before calculating principal components through the optimal weighted linear combination of food groups based on their correlation. Among all principal components, only a few that explain the most variation are retained for subsequent analysis. However, when the relationship between dietary patterns and demographic characteristics (e.g., age, income) is the focus, a posteriori exploratory analysis called Focused Principal Component Analysis (FPCA) can be applied [51]. The dietary patterns derived by FPCA are based on socioeconomic variables of interest and presented as concentric circles, where the center of the circle is a variable of interest. The distribution of different food group variables in the circle represents positive or negative correlations with the socioeconomic variable of interest in different colors or patterns. The smaller the radius, the stronger the correlation. The FPCA visualizes not only the relationship between the diet and a variable of interest but also the correlation between different food groups [51]. Like PCA, EFA reduces the dimensionality of food groups to a few factors with minimal loss of information. It decomposes each food group into common factors and a special factor: common factors are shared by all food groups, and special factors are unique to each food group. Each common factor represents a dietary pattern.

**Fig. 1:**
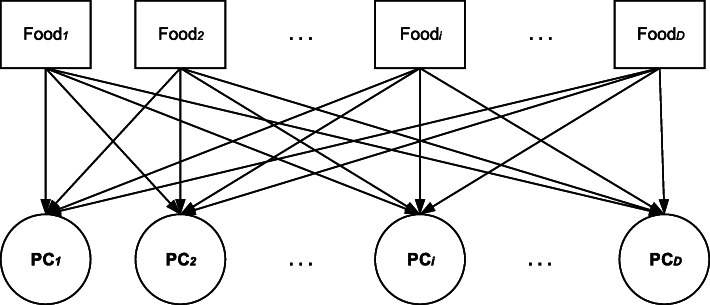
The principal component analysis with D food group variables. Each PC is a linear combination of D food groups and corresponds to a dietary pattern

When determining the number of principal components or factors to be retained, the three selection criteria that are typically used include 1) retaining factors with an eigenvalue greater than one, 2) the scree plot, and 3) the interpretable variance percentage [8]. The correlation coefficients between the principal component and the food groups are called factor loadings, and they reflect the importance of the food groups. The greater the absolute value of the factor loadings, the stronger is the correlation between the corresponding food groups and the principal components or factors. Therefore, the principal components or factors are named primarily based on the food groups retained by the selection criteria applied to the factor loadings. Owing to the similarity between PCA and EFA [10], only PCA is shown in Fig. 1.

Unlike EFA, confirmatory factor analysis (CFA) is seldom used in nutritional epidemiology [52]. However, CFA can impose statistical tests on the factor structure and factor loadings of food groups and determine the number of factors and food groups contributing significantly to those factors [2, 8]. In the past, CFA was applied as a second step to verify the goodness of fit and reproducibility of the factor structure of dietary patterns after PCA or EFA in the first step [9, 53, 54]. However, it remains uncertain whether the results are better than those obtained only with EFA [54]. Therefore, several studies have used CFA as a one-step approach to replace PCA or EFA [52, 55]. The advantage of CFA is that a latent variable model can be specified and tested, and additional priori knowledge can also be incorporated into the model [55].

#### Advantages

These methods describe the population’s variation in dietary intake and evaluate the overall quality of the diet. The resulting unrelated patterns capture the different dietary traits in the population and can be used directly as covariates to construct statistical models with health outcomes. Thus, they are more interpretable and meaningful than traditional methods that use a single nutrient or food. Moreover, some studies have found that several major dietary patterns derived by these methods show some reproducibility in different populations [56–59].

#### Disadvantages

These methods have subjectivity in selecting food groups, determining the number of principal components or factors, selecting which foods have large factor loadings, and the patterns’ nomenclature. In classic PCA and EFA, each principal component or factor is a linear combination of all the food groups, which creates interpretive difficulties. The extracted dietary pattern can only explain part of the total variance of the food groups; therefore, it only represents the optimal model related to the explainable variance. Although other patterns may provide important information, they may not be retained by the selection criteria, and thus this important information is ignored [60]. In response to the question, “Which dietary patterns have the most predictive capability of a disease?” both PCA and EFA are unable to give an accurate answer. Additionally, FPCA can only determine the correlation between one lifestyle and dietary patterns, but dietary patterns may have strong interactions with many lifestyle characteristics simultaneously, and it is difficult to separate dietary pattern effects from other lifestyle effects [61, 62].

#### Commonly available software and packages

The “proc princomp” and “proc factor” commands in SAS. The “survival” and “psych” packages in R. The “pca” and “factor” commands in STATA. SPSS.

### Clustering methods

In PCA and EFA, the food items collected are pre-grouped to the extent that they are correlated with one another, and each person receives a score for each dietary pattern. Therefore, these methods can help us understand which foods are eaten simultaneously among the population and the relationships between dietary patterns and health outcomes. Both PCA and EFA are considered methods for “clustering” the food groups [10]. However, clustering methods can classify individuals into different groups based on their characteristics [63]. The dietary differences of individuals among different groups can be compared, and the characteristics of dietary patterns can be described by calculating the average intake level of different food groups within each group. Groups can also be compared with a specified control group to explore the risk of disease outcomes in different groups. In the study of dietary patterns, the clustering methods are summarized in the following two categories.

#### Traditional cluster analysis (TCA)

**Fig. 2:**
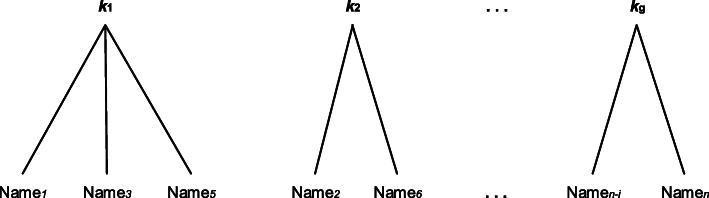
The k-means clustering with n individuals and g clusters. The individuals with similar dietary characteristics are assigned to one cluster

In nutrition research, TCA is based on the use of individual dietary characteristics to separate people into mutually exclusive clusters. One cluster represents a dietary pattern, with the individuals only belonging to one cluster [10], which is also called “hard” clustering. Before clustering, all the selected dietary variables (nutrients, food, or both) must be standardized to prevent variables with large variances from disproportionately affecting the clustering results [8]. The analyst needs to select the measure of similarity in individual dietary intakes, such as the Euclidean distance, Mahalanobis distance, and similarity coefficient, of individual dietary intakes. Clustering algorithms are then used to place similar individuals into the same category, and dissimilar individuals are dispersed as far as possible [10]. There are many clustering algorithms in TCA; three are commonly applied in dietary pattern analysis: k-means clustering, Ward’s minimum-variance method, and flexible-beta clustering [2, 64]. Figure 2 shows the main principles of TCA using k-means clustering as an example for comparison with FMM.

The k-means clustering algorithm is the most commonly used algorithm [65]. It has the advantages of low computation complexity, fast calculation speeds, and suitability for large samples. However, the k value often needs to be pre-specified by the researcher. Ward’s minimum-variance method is a hierarchical clustering algorithm, and all of the calculations required for the clustering process occur at once [10]. Even if the number of clusters changes, recalculation is not required. However, the calculation is complex and slow, making this method unsuitable for large samples [66]. The flexible-beta clustering algorithm is an agglomerative hierarchical clustering algorithm with a specified parameter and robust results [64, 67]. This algorithm introduces a new parameter *β* in the distance formula, for which the selected values are usually − 0.25 and − 0.50 [67]. However, there are only a few examples applying this method to the analysis of dietary patterns.

There is no singular method for identifying the number of clusters or an appropriate clustering algorithm [68, 69]. One approach is to combine several methods, that is, based on factor analysis, the appropriate k value and a reasonable initial cluster center are identified by hierarchical clustering to minimize the influence of subjective judgment on the clustering results [68, 70]. The other approach is the optimal clustering method, in which several different k values are tried, and quantitative indicators for these k values are compared to select the optimal value of k [8, 71]. The selection of the clustering algorithm mainly depends on the stability of the clusters and their reproducibility, which are often evaluated by the split-half cross-validation method or classifier [64, 72]. The most appropriate clustering algorithm is the one with the highest reproducibility and stability.

##### Advantages

Distinct subgroups of individuals can be identified according to their dietary characteristics, and everyone belongs only to one specific dietary pattern group. Thus, the relationship between dietary pattern subgroups and health outcomes or other characteristics can be examined, and the subgroup at nutritional risk can also be identified. The results are also highly intuitive, and a dendrogram can be drawn to show the clustering process and results visually.

##### Disadvantages

There are, however, a few drawbacks: first, each individual is assigned a cluster with a probability of 1 or 0, without considering the uncertainty of individual classification [73]. Second, the researcher is required to make several subjective decisions, such as the selection of the food groupings, clustering algorithms to determine the similarity of individuals, initial values, and the number of clusters. Although some relatively objective methods for selecting clustering algorithms and the number of clusters exist, the reproducibility of results cannot ensure their validity [64]. Third, there is no convenient method for comparing different clustering criteria [74]. Finally, the use of a control group and the unequal sample size of different clusters will limit the power of the statistical analysis [75].

##### Commonly available software and packages

The “proc cluster” command in SAS. The “psych” packages in R. The “cluster”, “clustermat” and “cluster kmeans” commands in STATA. SPSS.

#### The finite mixture model (FMM)

**Fig. 3:**
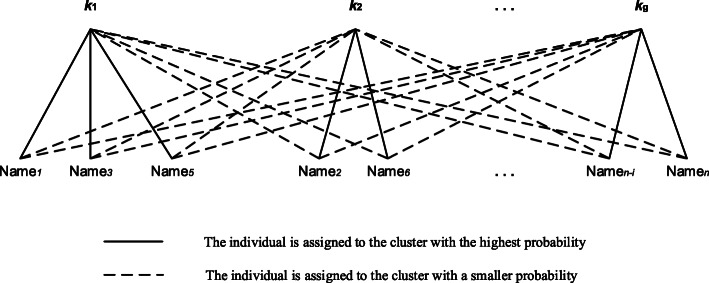
The finite mixture model with n individuals and g clusters. Each individual is only assigned to the cluster with the highest probability

The FMM is a clustering method based on a latent variable model [73, 76]. It measures classification uncertainty by calculating a posterior probability of different clusters based on given data; it is also called “soft” clustering [73, 74]. The FMM assumes that the observed dietary data will be decomposed into a mixture distribution representing a finite sum of different food consumption probability distributions. Each distribution represents an unobserved cluster corresponding to a dietary pattern [73]. Through FMM, each individual’s posterior probability is calculated for each cluster; the individual is then assigned to the cluster with the highest posterior probability (Fig. 3). The posterior probability can measure the uncertainty of assigning individuals to different clusters. The process is similar to a k-means algorithm, but the probability of each individual assigned to each cluster is used for classification.

Because FMM has many parameters, large samples are required. Thus, a restricted mixture model is proposed that reduces the number of parameters and is suitable for small- to moderately-sized samples [77]. The FMM method can also be used to classify the population according to the factor scores from factor analysis, also called a two-step classification, combining the advantages of both [76]..

##### Advantages

The choice of *k* values or models can be transformed into a statistical model selection problem. The final model is then identified according to the maximum Bayes Information Standard after the FMM is fitted by setting different k values or imposing different restrictions on covariance matrixes [78]. The FMM is more flexible than TCA as it can account for the within-class correlation between variables [63], allow the variances of food consumption frequencies to vary within and between clusters, and enable covariate adjustment for food intake (e.g., energy intake and age) simultaneously with the fitting process [74, 77].

##### Disadvantages

The observed data may violate the distribution hypothesis, especially when there are many zero values so that the flexibility of the FMM cannot be fully realized. Although there are some common methods for dealing with zero values, the need to deal with zero values increases the model’s complexity, as does the high number of parameters to be estimated [63]. Its algorithm for estimating parameters still has flaws such as sensitivity to the initial value, convergence to local extremum, and slow convergence speed.

##### Commonly available software and packages

The “flexmix” and “mclust” packages in R. The “proc fmm” and “proc lca” commands in SAS. The “fmm” and “gllamm” commands in STAT A. Latent GOLD. Mplus.

### The Treelet transform (TT)

Both PCA and FA are the most popular methods for identifying dietary patterns, but their qualitative interpretation is difficult and requires subjective judgment [79]. Additionally, cluster analysis fails to give numeric summary variables like factors or components. To overcome these limitations, the TT was developed to simplify the explanation of the factors while at the same time combining the advantages of PCA and the hierarchical clustering algorithm [79, 80].

**Fig. 4:**
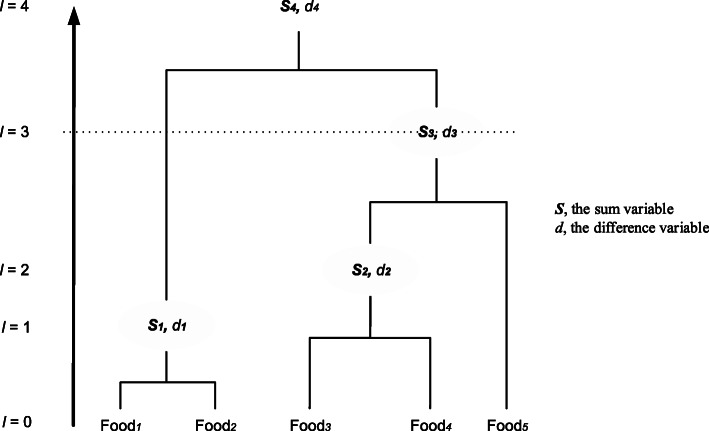
A cluster tree produced by the treelet transform with five food group variables. As the dashed line goes up, the cutting level moves away from the root, so the factor loadings become more sparse

Like PCA, TT produces a set of factors based on the food groups’ covariance or correlation matrix and introduces the sparsity hypothesis into the factor loadings. Consequently, only a few of the factor loadings of the food variable are non-zero, and others are all zero [79, 80], simplifying the explanation of factors. In nutrition epidemiology, the sparsity hypothesis holds if some foods are consumed independently of the foods included in the dietary patterns, or there is no variation in the population [81]. In the first layer of the cluster tree, the method identifies the two variables with the highest correlation among all the food groups and performs a PCA to produce two factors. The first factor is called the sum variable representing the weighted average of the largest variance, and the second factor is called the difference variable representing the orthogonal residual factor. Only the sum variable is retained in the cluster tree to repeat the algorithm above until each food variable is included in the cluster tree (Fig. 4).

After the cluster tree is built, it is “cut” at a given level to produce a high variance factor describing the relevant food groups. Unlike PCA, TT requires a researcher to cut the cluster tree at a given level and then extract the factors based on the factor variance at that level. After the retained number of factor k is determined, the optimal cut level is identified by 10 cross-validations [79, 80]. When the cutting level increases, the optimal cutting level corresponds to the inflection point when the cross-validation score (i.e., the mean of the k-factor variance sum) is no longer increased [79, 80]. Additionally, the TT analysis is repeated at ±3 levels of optimal cut levels to evaluate the sensitivity of different cut levels [80].

#### Advantages

Like PCA, the TT produces a set of factors, but each factor involves only a small percentage of food groups that simplify dietary patterns. When sample sizes are small, and the data are sparse with unknown groupings of correlated or collinear variables, TT is remarkably suitable for dimension reduction and feature selection before regression and classification [80]. Moreover, TT visualizes the results by constructing a hierarchical clustering tree for all variables, making the final results easily interpretable.

#### Disadvantages

Choosing the cutting level of the cluster tree before extracting factors requires subjective judgment. When the cutting level is close to the root, more variables are contained in the factors, and the difficulty of interpretation also increases. As the cutting level gradually moves away from the root, the factor loadings become sparse, and the factors become easily interpretable; however, the diet’s complexity cannot be reflected by some food groups [82]. If food groups are all associated in a meaningful way, or the correlation of some foods is too strong, then the sparsity hypothesis may not hold [81]. Additionally, it remains debatable whether TT is superior to other methods in exploring the relationship between diet and health outcomes [79, 83].

#### Commonly available software and packages

The “treelet” package in R. The “tt” commands in STATA.

## Hybrid methods

Investigator-driven methods are hypothesis-oriented approaches, which neither reflect the overall dietary patterns nor consider the relevant relational structure of nutrients. In addition, data-driven methods do not consider any priori professional knowledge on health outcomes; therefore, both methods are nonoptimal for identifying which dietary patterns can best predict disease risk [84]. Hybrid methods combine these two classes of methods to identify dietary patterns.

### Reduced rank regression (RRR)

**Fig. 5:**
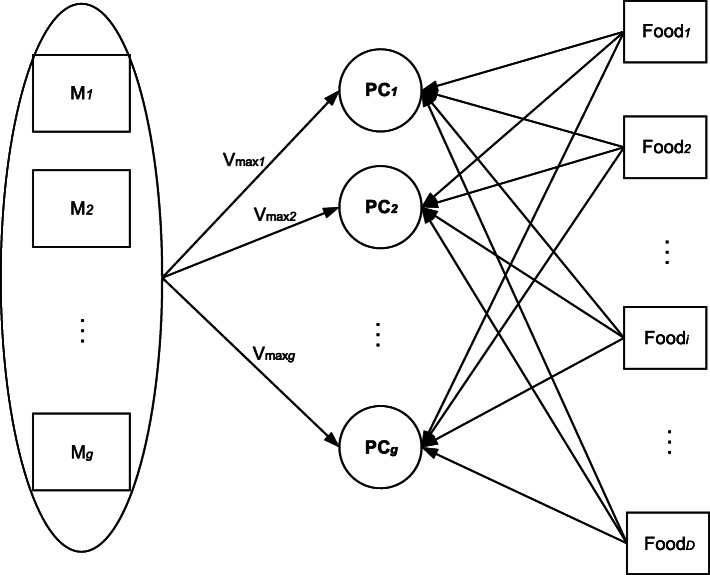
The reduced rank regression with D food group variables and g intermediate response variables (M). Each PC corresponding to a dietary pattern is a linear combination of D food groups which explaining as much variance (V_max_) in M as possible. D is larger than g

The RRR method considers both the disease-relevant variation in dietary intake and available dietary data in deriving dietary patterns [85, 86]. Specifically, RRR selects a set of disease-related variables, known as intermediate response variables, based on priori knowledge, then derives dietary patterns based on the existing dietary data [85]. Its mathematical foundation and method of deriving dietary patterns are similar to those of PCA. However, unlike PCA, which explains as much variance in food groups as possible, RRR identifies linear combinations of food groups that can explain the maximum variance in intermediate response variables (Fig. 5). Both RRR and PCA produce components, which are based on the number of food variables and response variables, respectively. Therefore, RRR can be considered a PCA of intermediate response variables. The key to RRR is the choice of intermediate response variables, which should be related to both the disease of interest and the diet. The commonly used response variables include nutrients, biomarkers, contaminants, and intermediate phenotypes, or a combination of several kinds of them, in which nutrients and biomarkers are the most widely used [84].

A method similar to RRR is partial least squares (PLS), a regression model of multiple predictor variables on multiple response variables [85]. The PLS method uses the covariance matrix of multiple intermediate response variables and multiple food groups to produce the factors; it is regarded as a compromise between PCA and RRR [85, 87, 88]. It not only contains information about intermediate response variables but also enables the discovery of important disease-related dietary intake, in which some nutrients may not be included in the intermediate response variables [87].

#### Advantages

RRR uses both priori information for defining appropriate intermediate response variables and the existing data. Thus, it combines the respective characteristics of investigator- and data-driven methods. This method includes the pathophysiological pathway linking dietary patterns with the disease [89]; therefore, the correlation between dietary patterns and disease outcomes may be more robust in RRR than in other methods, and the importance of dietary patterns in the etiology of diseases can be better studied [9]. The effect of dietary patterns on disease risk can be described and explained by changes in biologically important intermediate variables [8]. The relationship between dietary patterns and diseases of interest can be reproduced across studies [50, 84].

#### Disadvantages

The underlying disease development mechanisms need to be identified, as they are the effective intermediate response variables. If the information for disease development is absent, then RRR cannot be used [9, 90]. Additionally, there is no best way to choose the most appropriate intermediate response variables, and the commonly used method is based on priori information [8]. For many chronic diseases, complex interactions in metabolic pathways can link dietary intake to disease, but it is unclear whether the biomarkers of one metabolic pathway used in RRR are more effective than other potential metabolic pathways. Additionally, relying solely on the information of selected intermediate response variables to derive dietary patterns may lead to the omission of those dietary patterns related to nutrients in the disease’s biological pathways but are not included in the intermediate response variables [91].

#### Commonly available software and packages

The “proc pls” commands in SAS. The “rrr” and “rrpack” packages in R. The “rrr” commands in STATA.

### Data mining (DM)

DM can extract hidden information from large databases, allowing researchers to focus on the most important information in the data [92]. This method uses various data analysis tools to derive dietary patterns and help researchers make decisions [93, 94]. As one of the most important classification tools in DM, decision tree induction can be regarded as a clustering algorithm that makes full use of interesting health outcome. There have only been a few studies using this method in nutritional epidemiology until now [9, 94–96].

**Fig. 6:**
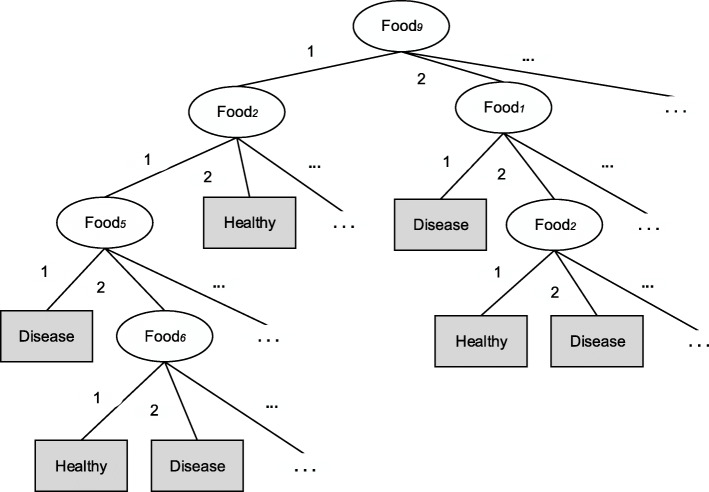
The decision tree generated by the C4.5 algorithm

Decision tree induction is also known as a classification and regression tree [9]. The main idea is to build a decision tree through a set of known training data and then use the established decision tree to predict new data sets. Establishing a decision tree can be regarded as the process of generating data rules, and the most classic algorithm is C4.5 [97]. This algorithm first pre-processes the selected food group variables by discretizing variables (e.g., expressing them as the frequency of food consumption). The classification result of interest is the health outcome. Then a “best” food group is selected as the root node of the decision tree and split according to its value to produce different subsets (“best” means that as far as possible, all individuals in the subset have the same outcome after splitting the data). The above procedures are then repeated on the subsets until the outcome of all individuals in each subset is the same. Each subset is called a leaf node, which constitutes the final decision tree (Fig. 6). A classification rule is a path from the root node to a leaf node associated with health outcomes. In the dietary study, the C4.5 algorithm needs to be run for all the combinations of different numbers of food groups to produce hundreds of classification rules. Repetitive and meaningless rules are deleted. The reserved rules correspond to dietary patterns. The intensity and direction of a food group’s association with diseases can be identified by comparing rules for which the only difference is the food group. Additionally, some other DM methods, such as random forest, artificial neural networks, and Naïve Bayes Classifiers, have also been used to analyze the relationship between dietary patterns and diseases [94, 95, 98], but they are all belongs to clustering algorithms and less common in nutritional epidemiology, so they are not introduced in more detail.

#### Advantages

When there is obvious heterogeneity in the dietary behavior of a population, DM can be used to reveal such heterogeneity and develop personalized preventive measures; the extent to which dietary components or patterns affect the course of the disease can also be identified [93]. It is also particularly useful in identifying disease risk based on a combination of known food groups and other non-dietary confounders [9]. Lastly, decision tree analysis can generate new hypotheses without priori assumptions or potential risk factors [99].

#### Disadvantages

If many classification rules are generated in the DM process, the selection of meaningful rules will require considerable professional knowledge. Rules containing many variables can be long and complex even if they are meaningful, making it difficult to translate them into simple health information. Additionally, one key variable can dominate the model; therefore, misclassification is more likely to occur with DM than with other methods [94].

#### Commonly available software and packages

The “proc split” and “proc hpsplit” commands in SAS and SAS/EM module. The “RWeka” and “rpart” packages in R. The “chaid” and “crtrees” commands in STATA. WEKA. SPSS.

### Least absolute shrinkage and selection operator (LASSO)

**Fig. 7:**
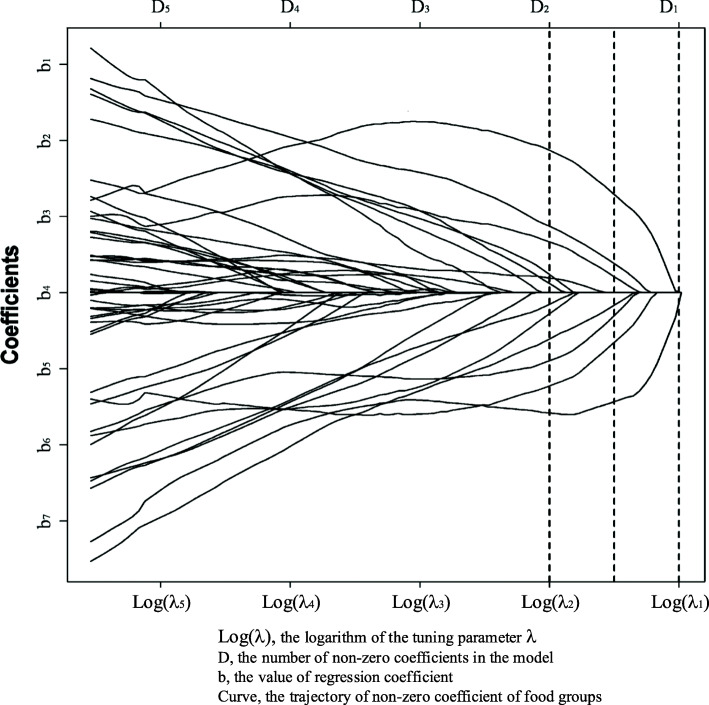
The least absolute shrinkage and selection operator. The number of points at which the dashed line intersects the curve represents the number of nonzero coefficients D. The smaller λ, the larger D

The LASSO model is a regression-based method that penalizes the regression coefficients’ absolute value so that the coefficients in the overall regression are shrunk [100]. Under the constraint that the sum of the absolute values of the regression coefficients is less than a constant, the sum of the squares of the residuals is minimized to obtain a sparse model in which some regression coefficients are shrunk to 0 [100]. Lasso’s complexity is controlled by the model tuning parameter λ; the greater the λ, the greater is the penalization of the model, resulting in a model with fewer variables. The LASSO model is hence a form of automatic feature selection. While identifying the dietary patterns, LASSO is directly applied to the defined food groups to predict health outcome [101]. Different λ results in different numbers of food groups with a non-zero coefficient selected into the model (Fig. 7). Cross-validation is used to select λ, which forces some coefficients of the food groups to zero and, hence, selecting food groups with non-zero coefficients [101]. The λ parameter is determined by the rule of minimum mean cross-validation error or one standard error. The selected food groups are then regarded as the dietary pattern.

#### Advantages

LASSO considers the outcome variable when deriving dietary patterns, thus achieving higher prediction accuracy. As a shrinkage method, the LASSO model selects for a subset of food groups to predict outcome that result in a more interpretable and relevant set of food groups.

#### Disadvantages

LASSO is still less applied in dietary pattern analysis, so its validity and reproducibility need to be confirmed in future studies. In addition, whether LASSO is superior to other dietary pattern methods in exploring the relationship between diet and health outcomes is yet to be verified.

#### Commonly available software and packages

The “glmnet” package in R. The “lassopack” commands in STATA.

## Compositional data analysis (CODA)

Usually, changes in one dietary component are accompanied by compensatory changes in others if the total energy intake is kept constant [5]. Therefore, dietary data can be regarded as compositional, that is, the data can also be referred to as compositional data [102, 103]. Compositional data can be used in analyzing the relative importance of food consumption and have great potential in dietary pattern analysis [5].

In the case of compositional data, *x* is a positive vector of D parts (*x* = [*x*_1_, *x*_2_, …*x*_*D*_]) and usually is a closed-form expression (proportions or percentages). Every composition *x*_*i*_ represents relative information that describes the parts of the whole. The mathematical difficulties inherent in compositional data have hampered their wider use [104]. Therefore, a method called compositional data analysis (CODA) [104] has been proposed; the method uses log-ratio coordinates to transform compositional data into a form that can be analyzed using standard multivariate statistical analysis. Owing to the compositions’ proportional nature, the only valid function of compositional data is composed of the ratio of different parts [102, 104]. There are three widely used transformation methods for log-ratio coordinates: additive log-ratio (alr), centered log-ratio (clr), and isometric log-ratio (ilr) transformations. In alr, each of the first D-1 parts is divided by the final part, but the transformation is not orthogonal; therefore, the rationality of statistical analysis cannot be guaranteed. The clr method can solve this limitation by dividing each part by the geometric mean of the D parts [105]; however, the sum of those clr variables is zero, meaning that perfect collinearity exists [106], which can be solved by the ilr transformation [107]. The ilr transformation preserves the original mathematical properties and geometric features; therefore, the rationality of directly applying the classic statistical method is ensured. Compositional data analysis has been applied in health research only recently, and there is less research on the relationship between dietary patterns and health [5]. There are three approaches to building the ilr transformational variables for dietary pattern analysis: compositional principal component coordinates, balance coordinates, and principal balances (PBs).

### Compositional principal component coordinates

Due to the constant sum and possible nonlinearity in compositional data, directly applying the traditional PCA will likely result in many problems [5]. Thus, Aitchison extended standard PCA to compositional data [105]. The main idea is that the standard PCA is applied to the clr transformed covariance matrix to extract the principal components called PC coordinates. It can be proved that PC coordinates satisfy all the ilr transformation conditions and are equivalent to ilr coordinates. The first few PC coordinates explaining the most variance in dietary intake can be used for studying the relationship between dietary patterns and health outcomes.

### Balance coordinates

The use of PC coordinates can be regarded as a data-driven ilr transformation, but it can also be a priori-driven based on the researcher’s questions or interests. In epidemiology, a priori-driven ilr transformation is calculated mainly by easily explainable balance coordinates [103, 108] representing the relationship between different groups of parts. To build balance coordinates, sequential binary partition (SBP) is used to divide the complete composition of D parts into two groups of parts successively in a hierarchical manner: one part for the numerator and the other for the denominator. Similarly, each of the two groups is again split into two new groups to create the new balance coordinate and so on until step D-1, when only a single part is left in each group. Then, D-1 different ilr balance coordinates are produced [108]. Each set of balance coordinates corresponds to a dietary pattern. Positive coordinates indicate that the numerator has a relatively high weight, and negative coordinates indicate that the denominator has a higher weight.

To enhance the interpretation of the analysis, SBP can be constructed based on the purpose of the study [102, 109]. For example, if the research aims to extract dietary patterns, ilr balance coordinates can be constructed according to natural or artificial clustering of different foods or nutrients in groups. Thus, balance coordinates are not data-driven and mainly focus on the research questions, unlike hierarchical clustering analysis. Since the total variance of the complete composition is decomposed into D-1 parts and the balance coordinates are independent of each other, all D-1 balance coordinates must be included as explanatory variables in the model simultaneously [5]. Balance coordinates can be visualized through a tree diagram, called the CoDa-dendrogram or the balance dendrogram, which is also a tool for describing the whole process of SBP [5, 110].

### Principal balances

**Fig. 8:**
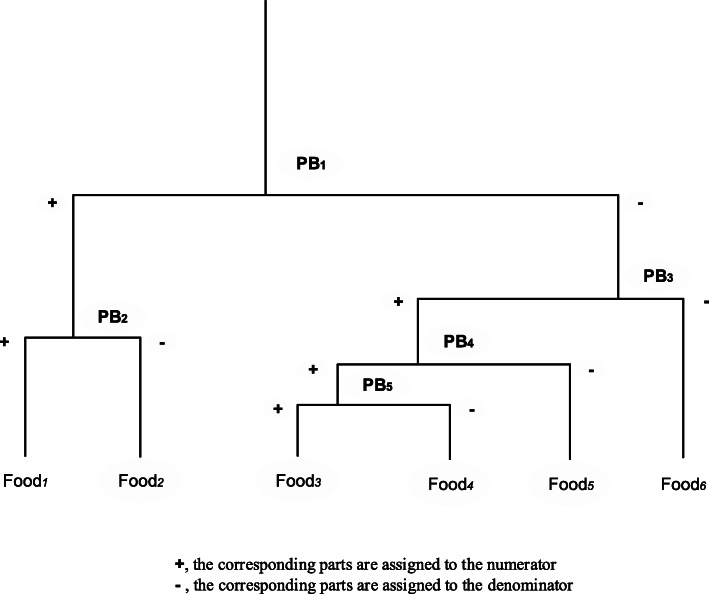
CoDa-dendrogram of PBs with six food group variables. Each PB corresponds to a dietary pattern. The closer the contact point is to a food, the more of that food is relatively more abundant

Principal balances are data-driven balance coordinates that can not only concentrate a large proportion of the total variance in a few coordinates but are also convenient for comparing groups of parts in the numerator and the denominator [109]. The first PB is the balance coordinate that maximizes the explained variance. The kth PB maximizes the remaining variance and is orthogonal to the previous k-1 PBs. All the PBs or the PBs with the highest variance can be used for subsequent analysis. In the CoDa-dendrogram, PBs are ordered by the variance of the balance, which are different from balance coordinates ordered by the sequence of the partitions [109]. A CoDa-dendrogram of PBs is shown in Fig. 8.

The optimal algorithm of PBs is an exhaustive search of all possible SBPs [109]. If data are highly dimensional, the computer space and time required will be large and long, respectively, so that using PBs on a personal computer becomes difficult. At present, suboptimal but faster algorithms have been proposed to search for PBs, such as the new constrained PCs algorithm and Ward’s cluster method; the proposed methods produce PBs whose variances are slightly lower than those obtained by optimization algorithms and are more applicable for high-dimensional compositional data [109].

### Advantages

These three coordinates—compositional principal component coordinates, balance coordinates, and PBs—can extract compositional information in dietary patterns for further direct application of classic multivariate statistical methods. The results emphasize that any dietary pattern is a balance between different intakes of food. When the relationship between dietary patterns and health outcomes is studied, the results can be interpreted as the effects of increasing the intake of some foods and reducing the intake of other foods proportionally on health outcomes. Therefore, considering food intake as compositional data is more consistent with the intuitive concept of dietary patterns and the practice of dietary recommendations. The first few PC coordinates and PBs can explain a large proportion of dietary intake variation. Additionally, balance coordinates and PBs are more easily explainable than PC coordinates, and they can be depicted as a CoDa-dendrogram.

### Disadvantages

Like PCA, each PC coordinate contains all the food groups, which complicates the explanation of the results, and factor loadings need to be recalculated for each application to different data sets [5, 105]. The balance coordinates are an investigator-driven method requiring sufficient priori knowledge to provide SBP, meaning that the subjectivity of SBP is inevitable. Especially when D is large enough, the selection of SBP will become difficult, or there will be more than one SBP. Additionally, most of the total variation cannot be explained by a few balance coordinates [5]. Finally, for a large number of zero values in the compositional data, especially absolute zero values, no method works well [111].

### Commonly available software and packages

The “coda.base,” “compositions,” “robCompositions,” and “zCompositions” packages in R. Stand-alone programs such as “SparCC” and “CoDaPack.”

## Conclusion

With the development of nutritional epidemiology over the past decades, there is extensive research on dietary patterns describing the features of dietary behavior or habits and explaining the relationship between diet and diseases [2]. Moreover, there is growing evidence that food-based dietary patterns are a better way of reducing cardiovascular disease, diabetes, and obesity than single dietary components, total fat, and calories [112]. Previous reviews have already introduced several classic methods of deriving dietary patterns, mainly focusing on dietary quality scores, PCA, FA, TCA, and RRR. However, other methods of identifying dietary patterns are rarely or never reviewed [2, 3, 7, 9, 10]. This paper provides an updated overview of the methodological aspects of various methods and briefly introduces their underlying concepts, advantages and disadvantages, and the software available for their implementation. These methods describe and explain potential complex eating behaviors from different perspectives. They aid researchers in studying the relationship between diet and diseases more comprehensively.

Dietary quality scores mainly aim to evaluate the quality of the overall diet and test the validity of dietary guidelines or recommendations [9, 13]. While MDS, HEI, AHEI, and DASH are especially recommended to predict disease risk, only the Mediterranean diet has been proven to reduce disease risk in both observational studies and randomized controlled trials [6, 41]. Data-driven methods are especially important for identifying the priorities of nutritional interventions and exploring the health effects of different dietary habits [9]. However, they are often criticized for not considering priori knowledge about diseases, so they are preferred methods for performing an explorative analysis [87]. Both PCA and FA capture the interrelation between dietary components by creating principal components or factors, but they are not easy to explain. The TT can be regarded as a complementary method to PCA because it produces similar scores, which are easily interpretable as the patterns have no contributions from some foods or food groups. Nevertheless, the assumption of such scenarios is often hard to verify, and sometimes the relationship between TT-derived dietary patterns and the disease is different from that of previous results [83], probably because not all foods are included in the score calculation, and the patterns fail to reflect the real complexity of diet intake. The main advantage of TCA is that it assigns each individual a specific dietary pattern subgroup, which is difficult for PCA, FA, and TT; thus, individualized dietary advice can be provided.

Another clustering method is FMM, which can calculate the probability of each individual assigned to each category, and the covariate adjustment is considered in the fitting process. However, it is still not as widely used as TCA, probably because of the requirements for distribution, the model’s complexity, and the need for more statistical expertise. Furthermore, FMM does not consistently give much better clustering results than the k-means algorithm at the cost of increasing model complexity [63]. None of these data-driven methods consider the health outcome when deriving dietary patterns and they are data- and population-specific; therefore, the results do not adequately explain the relationship between diet and diseases and have limited reproducibility.

The RRR method makes full use of a priori knowledge of biological relations to identify the dietary patterns with significant influence in the etiology of disease [85, 113] and is particularly useful in deriving dietary patterns related to given diseases and is reproducible across populations [50]. However, its application is limited to only diseases with adequate priori knowledge of intermediate response variables. Unlike RRR, the DM and LASSO methods use only one outcome variable at a time to identify dietary patterns. However, DM divides individuals into distinct subgroups similar to clustering algorithms to predict outcomes. It can identify which subgroups are at risk of the disease and explore new patterns of various diet and non-diet combinations. The LASSO model uses food groups to predict outcomes directly instead of constructing new underlying variables or dividing individuals into mutually exclusive subgroups. It performs prediction and variable selection simultaneously to build a sparse model.

Dietary intake data can also be regarded as compositional data with varying total diet intake among individuals [5, 114, 115]. Additionally, metabolic dysfunction can be caused not only by a lack of nutrients but often by an imbalance between nutrients [114]. Although compositional data are not a new concept, they have only recently been applied to nutritional epidemiology [5, 102, 103, 114]. In addition to being applied for dietary patterns, the CODA methods can also separate the specific effects of macronutrients from the generic effects of total calorie intake simultaneously [103]. Several new algorithms applying clustering methods (e.g., FMM and k-means clustering) or hybrid methods (e.g., RRR) to compositional data and compositional substitution models which will be possible to investigate specific food substitution have been proposed. However, they have not yet been applied in dietary pattern analysis [116–118].

Classical methods are useful in nutritional epidemiology, but we should not limit ourselves to them since emerging methods can provide improved results and new ideas to overcome the shortcomings and inapplicable problems of the classic methods under suitable scenarios. Therefore, emerging methods deserve more attention. Among them, CODA methods especially seem to hold great potential and promise for deriving dietary patterns and studying the relationship between diet intake and health outcomes differently. However, future research is needed to evaluate these emerging methods’ performance in terms of reproducibility, validity, and predicting different outcomes.

In summary, all methods of deriving dietary patterns can be used to answer different research questions. Hence, when conducting dietary pattern analysis, the first step is determining the problems to be solved and then selecting the appropriate method. If it is unclear which method is most suitable, combining multiple methods in the same study to produce complementary results and explanations is a good choice. However, there are many other problems that these methods cannot solve well, such as measurement errors (including large proportions of zeros), the interactions between dietary patterns and other non-dietary confounders, and the predictive effect of changes in dietary patterns on disease over time.

Some efforts have been made to address these problems. For example, some measurement error correction methods and new biomarkers of food intake have been developed for the measurement error [119, 120]; EPCA, DM, and LASSO can be used to explore the correlations between different diet and any other non-dietary confounders [51, 93]; and repeated measures of food intake in cohort studies can assess the changes in dietary patterns and provide stronger causality between food intake and disease [6, 41]. Additionally, we may also need to learn methods from other disciplines, including substitution models in behavioral epidemiology, pattern recognition methods in mathematics and computer science, and decision-making and optimization methods in operations research [2, 117]. Although increasing attention has been paid to dietary pattern research, it should be noted that dietary pattern research is not meant to replace single-nutrient research; the two types of research should coexist and complement each other.

We hope that this landscape review will help researchers in this field to understand and apply various methods effectively in practice and familiarize interested researchers outside the field with these methods. We also hope that methodological limitations will gain more attention and be improved to simulate new study ideas that may more accurately disclose the relationship between diet and health.

## Data Availability

Not applicable.
